# Antimicrobial Mechanisms of Leucocyte- and Platelet Rich Fibrin Exudate Against Planktonic *Porphyromonas gingivalis* and Within Multi-Species Biofilm: A Pilot Study

**DOI:** 10.3389/fcimb.2021.722499

**Published:** 2021-10-13

**Authors:** Fabio Rodríguez Sánchez, Tim Verspecht, Ana B. Castro, Martine Pauwels, Carlos Rodríguez Andrés, Marc Quirynen, Wim Teughels

**Affiliations:** ^1^ Department of Oral Health Sciences, Section Periodontology, Catholic University of Leuven and University Hospitals Leuven, Leuven, Belgium; ^2^ Department of Preventive Medicine and Public Health, University of the Basque Country, Bilbao, Spain

**Keywords:** biofilms, L-PRF, wound healing, antimicrobial mechanism of action, infection disease, oral microbiota

## Abstract

Leucocyte- and platelet rich fibrin (L-PRF) is an autologous biomaterial used in regenerative procedures. It has an antimicrobial activity against *P. gingivalis* although the mechanism is not fully understood. It was hypothesized that L-PRF exudate releases hydrogen peroxide and antimicrobial peptides that inhibit *P. gingivalis* growth. Agar plate and planktonic culture experiments showed that the antimicrobial effect of L-PRF exudate against *P. gingivalis* was supressed by peroxidase or pepsin exposure. In developing multi-species biofilms, the antimicrobial effect of L-PRF exudate was blocked only by peroxidase, increasing *P. gingivalis* growth with 1.3 log genome equivalents. However, no effect was shown on other bacteria. Pre-formed multi-species biofilm trials showed no antimicrobial effect of L-PRF exudate against *P. gingivalis* or other species. Our findings showed that L-PRF exudate may release peroxide and peptides, which may be responsible for its antimicrobial effect against *P. gingivalis*. In addition, L-PRF exudate had an antimicrobial effect against *P. gingivalis* in an *in vitro* developing multi-species biofilm.

## Introduction

Microorganisms associated with both caries and periodontal diseases are metabolically highly specialized and organized in multi-species microbial biofilms. A homeostatic balance usually characterizes multi-species biofilms under health conditions. Nevertheless, some highly specialized members of those communities can play an important role in provoking a dysbiosis and deregulation of the host immune response driven by several specific factors such as environmental stressors, inflammation and positive feedback loops. This may result in the destruction of periodontal tissues in susceptible individuals (Sanz et al., 2017).

Among the microorganisms isolated from patients suffering from periodontal pathologies, *Porphyromonas gingivalis* is the most commonly found (Davey, 2006; Brunner et al., 2010). This Gram-negative and obligate anaerobic bacterium produces several virulence factors that contribute to its pathogenicity (Tokuda et al., 1996; Jain and Darveau, 2010), for instance enabling the invasion of periodontal tissue and providing protection against the host defence (Lamont et al., 1995; Laine et al., 1997). In addition, the intra-oral presence of this microorganism has been identified as a risk factor for pulmonary infections, preterm delivery and low birth weight (Scannapieco, 2006; Offenbacher et al., 2006). Furthermore, their presence in atherosclerotic plaques was shown to increase the risk of myocardial infarction, and it was also isolated from dentoalveolar abscesses (Dymock et al., 1996; Sanz et al., 2020).

One of the most common periodontal causative treatments consists of subgingival debridement, and a surgical intervention for the more advanced types of periodontitis (Slots, 2017). Subgingival debridement, as defined during the first European Workshop on Periodontology (Lang and Karring, 1994), aims for the disruption and/or removal of the acquired biofilm so that (re)attachment of periodontal tissues to the root surface can occur (Drisko, 2001; Dentino et al., 2013). The clinical end points of periodontal debridement are, among others, the reduction of periodontal inflammation and probing pocket depths less than 5 mm (Laleman et al., 2017). Periodontal surgery would be recommended when the pockets are ≥ 5 mm, given that residual pockets of ≥ 5 mm are predictive for further attachment loss and tooth loss (Claffey and Egelberg, 1995; Heitz-Mayfield, 2005; Matuliene et al., 2008). Moreover, antimicrobial agents and antiseptic solutions are frequently prescribed as adjunctive to the initial non-surgical therapy (da Costa et al., 2017).

Recently, new tissue engineering techniques have been studied for regenerative procedures after non-surgical periodontal therapy (Shanbhag et al., 2019). Platelet concentrates have been used and studied to enhance and speed up wound healing by promoting recruitment, proliferation, and maturation of cells involved in tissue healing and regeneration (Boswell et al., 2012). Leucocyte- and platelet rich fibrin (L-PRF), a second-generation platelet concentrate, was introduced as an autologous biomaterial that serves as scaffold for regenerating cells. L-PRF is prepared from the patient’s own blood, without additives, and concentrates more than 80% of the platelets and more than 75% of the leucocytes of what is present in the initial blood sample (Dohan Ehrenfest et al., 2010). Different forms of L-PRF can be prepared. The L-PRF exudate is produced after compressing the L-PRF clots to get the L-PRF membrane, which is the most frequently employed L-PRF material (Castro et al., 2019a; Castro et al., 2019b).

L-PRF offers a continuous release of bioactive elements, such as growth factors (transforming growth factor β1, vascular endothelial growth factor, platelet-derived growth factor AB), cytokines (interleukin 1, 4 and 6) and bone morphogenic proteins (BMP 1, 2 and 9) that stimulate and protect the surgical site (Dohan Ehrenfest et al., 2009; Anitua et al., 2012; Li et al., 2013; Castro et al., 2019a).

Various studies have reported other biological properties of L-PRF, such as enhancing wound healing, diminishing post-operative pain and minimizing the risk of infection (Bi et al., 2020; Petrescu et al., 2021). Furthermore, recent research in this also reported an antimicrobial effect of L-PRF against key periodontal pathogens (Castro et al., 2019b). This study assessed the antimicrobial properties of L-PRF against the main periodontopathogens, grown on agar plates and in planktonic cultures. The authors concluded that an L-PRF membrane had a strong antimicrobial capacity, especially against *P. gingivalis.* The L-PRF exudate also caused a strong inhibition of *P. gingivalis* grown on agar plates. Moreover, L-PRF exudate decreased the numbers of viable *P. gingivalis* in a dose-dependent way (Castro et al., 2019b).

However, the mechanism behind the antimicrobial effect of L-PRF on *P. gingivalis* has not been fully understood yet. Current evidence suggests that platelets may play multiple roles in antimicrobial host defence (Yeaman, 2014). They generate oxygen metabolites, including superoxide, hydrogen peroxide and hydroxyl free radicals, capable of binding, aggregating, and internalizing microorganisms. In addition, platelets can also release an array of potent antimicrobial peptides (Blair and Flaumenhaft, 2009).

The primary aim of this study was to characterize the mechanisms involved in the antimicrobial effect of L-PRF exudate against *P. gingivalis*. The secondary aim of this study was to evaluate the antimicrobial effect of L-PRF exudate against *P. gingivalis* in a multi-species biofilm. The null hypotheses were postulated as (1) L-PRF exudate does not release peroxide or/and peptides that inhibit or decrease *P. gingivalis* growth, (2) L-PRF exudate has no effect on *P. gingivalis* in a pre-formed multi-species biofilm, and (3) L-PRF exudate does not release peroxide or/and peptides that inhibit or decrease the growth of *P. gingivalis* in a developing multi-species biofilm.

## Methods

### Blood Collection and L-PRF Preparation

L-PRF samples were obtained from the blood of one systemically healthy and non-smoker adult volunteer (27-year-old male) who had not taken any antibiotics for 6 months before the study. Blood was collected with sterile 9-mL silica-coated plastic tubes without anticoagulant (BVBCTP-2, Intra-Spin, Intra-Lock, FL, USA) and immediately centrifuged at 408 x *g* for 12 min with a table centrifuge (IntraSpin, Intra-Lock, Boca Raton, FL, USA). After centrifugation, the L-PRF clot was carefully removed from the tube and it was transformed into a membrane (1-mm in thickness) by gentle compression (KU Leuven Congres, 2018). The liquid released during the compression, also called L-PRF exudate, was stored at -80°C for further use. No complications were reported during blood collection.

### Bacterial Strains and Culture Conditions

The following bacterial collection was used: Aggregatibacter actinomycetemcomitans (ATCC 43718), Prevotella intermedia (ATCC 25611), Porphyromonas gingivalis (ATCC 33277), Fusobacterium nucleatum (ATCC 20482), Streptococcus mutans (ATCC 20523), Streptococcus sobrinus (ATCC 20742), Actinomyces naeslundi (ATCC 51655), Actinomyces viscosus (DSM 43327), Veillonella parvula (DSM 2008), Streptococcus oralis (DSM 20627), Streptococcus sanguinis (LM14657), Streptococcus gordonii (ATCC 49818), Streptococcus mitis (DSM 12643), Streptococcus salivarius (TOVE-R). These 14 bacterial species were maintained on blood agar (Oxoid, Basingstoke, UK) supplemented with 5 mg/mL hemin (Sigma, St. Louis, USA), 1 mg/mL menadione (Calbiochem-Novabiochem, La Jolla, CA, USA), and 5% sterile horse blood (E&O Laboratories, Bonnybridge, Scotland). Overnight liquid cultures were prepared in Brain Heart Infusion (BHI) broth (Difco, Detroit, MI, USA). Bacteria were cultured under anaerobic conditions (80% N_2_, 10% H_2_ and 10% CO_2_) in the case of P. gingivalis, Prevotella intermedia, Fusobacterium nucleatum, Actinomyces naeslundii, Actinomyces viscosus and Veillonella parvula, or under aerobic conditions (5% CO_2_) for Aggregatibacter actinomycetemcomitans, Streptococcus sanguinis, Streptococcus gordonii, Streptococcus salivarius, Streptococcus mitis, Streptococcus oralis, Streptococcus mutans and Streptococcus sobrinus.

### Characterization of the Mechanisms Involved in the Antimicrobial Effect of L-PRF Exudate Against *P. gingivalis*

#### Agar Plate Experiments

Antagonistic experiments were performed on blood agar plates (Difco, Sparks, MD, USA) and modified BHI agar plates (Alvarez et al., 2013) supplemented with 5 mg/mL hemin (Sigma, St. Louis, MO, USA) and 1 mg/mL menadione (Calbiochem-Novabiochem, La Jolla, CA, USA) using the spotting technique (Herrero et al., 2016). An overnight culture of *P. gingivalis* was centrifuged (1438 x *g*, 10 min) and adjusted to an OD_600 nm_ of 0.5 (~1x10^8^ CFU/mL). Next, 100 µL of this bacterial suspension was inoculated uniformly on both blood agar plates and modified BHI agar plates using a cotton swab. The agar plates were anaerobically incubated at 37°C for 1 h to dry the bacterial solution on the agar surface in advance of spotting. Mixtures consisting of L-PRF exudate combined (ratio 1:1) with phosphate-buffered saline (PBS) supplemented with horseradish peroxidase (HRP; 80 µg/µL; Sigma, St. Louis, MO, USA), PBS supplemented with trypsin-EDTA (0.1%; Gibco-Thermo Fisher Scientific, Grand Island, NY, USA) or PBS supplemented with pepsin from porcine gastric mucosa (128 µg/µL; Sigma, St. Louis, MO, USA) were prepared. The solutions were sterilized with a 0.2 µm pore size and 25 mm diameter (50/pkg) sterile syringe filters (Pall Acrodisc^®^ with Supor^®^ Membrane, Pall Laboratory, Port Washington, NY, USA). Afterwards, these mixtures were incubated aerobically at 37°C for 30 min to let the enzymes act upon the peroxide or peptides possibly present in the L-PRF sample. Subsequently, one drop of 10 µL of L-PRF exudate exposed to either HRP, trypsin-EDTA or pepsin was inoculated directly on top of the bacterial inoculum on the agar plates. HRP, trypsin-EDTA and pepsin had a final concentration of 40 µg/µL, 0.05% and 64 µg/µL, respectively. Ten µL of L-PRF exudate diluted in PBS (1:1) without these enzymes were used as *diluted* positive control. Another 10 µL of pure L-PRF exudate without PBS or any enzyme was considered as *undiluted* positive control. Ten µL of chlorhexidine 0.12% was also used as positive control. On the other hand, 10 µL of PBS was used as negative control. L-PRF exudate was inactivated following the protocol described by Soltis and co-workers (Soltis et al., 1979). Briefly, L-PRF exudate was heat-inactivated in a heat block at 56°C for 30 minutes. Immediately after that, the sample containing the *inactive* L-PRF was cooled down with tap water. Then, 10 µL of *inactive* L-PRF exudate was used as negative control.

After 48 h of anaerobic incubation (37°C), the inhibitory effect of the six different conditions was evaluated. A calibrated photograph was taken of the agar plates and the inhibition areas (IA) were measured and calculated with ImageJ^®^ software (Image Processing and Analysis in Java, 1.8.0_77).

#### Planktonic Culture Experiments

Antagonistic experiments using planktonic cultures were performed as follows. An overnight culture of *P. gingivalis* was adjusted to and OD_600 nm_ of 0.5 (~1x10^8^ CFU/mL), centrifuged (1438 x *g*, 10 min) and re-suspended in fresh double-concentrated modified BHI supplemented with 10 mg/mL hemin and 2 mg/mL menadione due to the future 1:2 dilution after adding the test and control substances. The modified BHI used in this experiment was the same as the one used in the agar plates experiments but without containing the agar. Afterwards, a sample was taken for a viability-qPCR analysis to determine the initial bacterial concentration. Successively, 150 µL of this bacterial suspension was added to seven different wells of a 96-well plate (150 µL/well). These 96-well culture plates were made of polystyrene, sterile and had a physically treated surface (Greiner CELLSTAR^®^, Kremsmünster, Austria).

Subsequently, two 150-µL L-PRF exudate solutions supplemented either with HRP (80 µg/µL) or pepsin (128 µg/µL) were aerobically incubated at 37°C for 30 minutes and then added to two different wells of the 96-well plate containing the bacterial suspensions. These two test groups had identically final concentrations as the ones used on the agar-plate assays (40 µg/µL for HRP and 64 µg/µL for pepsin). As a negative control, 150 µL of PBS was added to one of the seven wells containing the adjusted *P. gingivalis* overnight culture. A 150-µL solution of *inactive* L-PRF exudate was added to one of the seven wells containing the adjusted *P. gingivalis* overnight culture and was used as negative control. The heat-inactivation procedure was identical to the one described for the agar plate experiments (Soltis et al., 1979). Two different 150-µL solutions of chlorhexidine (0.12% and 0.2%) were respectively used as positive controls. Two different concentrations of chlorhexidine were used to compare them with the inhibition areas of the tested solutions and to eventually establish a relationship in means of inhibition power. Each of them was added to a different well containing 150 µL of the adjusted *P. gingivalis* overnight culture. In addition, 150 µL of L-PRF exudate was added to another well containing the adjusted *P. gingivalis* overnight culture as a positive control. Consequently, the positive control’s final concentration used in this planktonic culture assay was equivalent to the *diluted* positive control’s final concentration used on agar plates. All test and control solutions were sterilized with sterile syringe filters before any anaerobic incubation.

After 24 h of anaerobic incubation at 37°C, bacterial DNA was extracted from 90 µL of each condition and quantified by means of viability-qPCR in order to calculate differences in bacterial numbers, expressed as the logarithmic values of genome equivalents per millilitre (log_10_(Geq/mL). These differences were calculated by deducting the bacterial number of the initial inoculum from the bacterial numbers obtained for each condition (Δlog_10_(Geq/mL)). The enzymatic blocking effect on the growth inhibition of *P. gingivalis* exerted by the L-PRF exudate was contrasted against a control group containing L-PRF exudate without any enzymes.

### Effect of L-PRF Exudate Against *P. gingivalis* in a Multi-Species Biofilm

#### Multi-Species Biofilm Preparation

A 14-species community was pre-formed using a BIOSTAT^®^ B TWIN bioreactor (Sartorius, Germany). 750 mL of modified BHI broth was added to the reactor vessel together with 5 mg/mL hemin, 1 mg/mL menadione and 200 μL/L antifoam Y-30 (Sigma, St. Louis, USA). The medium was pre-reduced over 24 h at 37°C by bubbling 100% N_2_ and 5% CO_2_ in the medium under continuous stirring at 300 rpm. The pH was set at 6.7 +/− 0.1. After 24 h, each overnight culture of *S. sanguinis, S. gordonii, S. mitis, S. oralis, S. mutans, S. sobrinus, A. viscosus, A. naeslundii, P. intermedia, P. gingivalis, F. nucleatum, A. actinomycetemcomitans, S. salivarius, and V. parvula* were adjusted to an OD_600 nm_ of 1.4 and added to the bioreactor vessel. During the first 48 h, the medium was not replaced. After that, the medium was replaced at a rate of 200 mL/24 h (Herrero et al., 2016). The effect of the L-PRF exudate on biofilm composition was evaluated using both during biofilm formation and using a pre-formed biofilm.

#### Pre-Formed Multi-Species Biofilm Experiments

Briefly, a bioreactor-derived 14-species co-culture was diluted 1:10 in modified BHI. Next, a sample was taken to determine the initial concentration of each species by means of a viability-qPCR. The diluted (1:10) 14-species community was inoculated in a 24-well plate (1 mL/well), followed by anaerobic incubation (37°C, 24 h). These 24-well culture plates were made of polystyrene, sterile and had a tissue culture treated surface (Greiner CELLSTAR^®^, Kremsmünster, Austria). Afterwards, the supernatants were removed from all wells and the baseline bacterial concentrations in the pre-formed biofilm were determined by means of viability-qPCR after detaching the biofilm from one well as described below. Subsequently, 1 mL of a solution containing L-PRF exudate and modified BHI in a 1:1 ratio was added to the test group’s wells, followed by anaerobic incubation (37°C, 24 h). The modified BHI used in this experiment was the same as the one used in the agar plates experiments but without containing the agar.

The negative control was obtained by the addition of 500 µL modified BHI combined with 500 µL L-PRF exudate. L-PRF inactivation was achieved by heating the sample containing the L-PRF exudate in a heat block at 56°C for 30 min. Immediately after that, the sample containing the *inactive* L-PRF was cooled down with tap water and was used as negative control (Soltis et al., 1979). Another negative control was obtained by the addition of 1 mL of modified BHI. The positive control was a solution of 500 µL modified BHI mixed with 500 µL chlorhexidine 0.12%. All test and control solutions were sterilized with sterile syringe filters before any anaerobic incubation.

After 24 h of anaerobic incubation (37°C), supernatants were removed. Subsequently, treated biofilms were detached by adding 1 mL trypsin-EDTA 0.05% for 5 min (37°C), then centrifuged (6000× *g* for 5 min) and re-suspended in 500 µL PBS. Afterwards, bacterial DNA was extracted from 90 µL of these samples and quantified by means of viability-qPCR in order to calculate differences in bacterial numbers, expressed as the logarithmic values of genome equivalents per millilitre [log_10_(Geq/mL). These differences were calculated by deducting the baseline bacterial numbers of the pre-formed biofilm for each bacterial number of the initial inoculum from the bacterial numbers obtained for each condition (Δlog_10_(Geq/mL)]. The inhibitory effect of L-PRF exudate on *P. gingivalis* was compared to the effect of inactivated L-PRF exudate and modified BHI as negative controls and to the effect of chlorhexidine 0.12% as positive control.

#### Developing Multi-Species Biofilm Experiments

For developing multi-species biofilm experiments, 500 µL of the same 14-species community used in the pre-formed multispecies biofilm experiments (diluted 1:5 in modified BHI) was combined with 500 µL of L-PRF exudate in the wells of a 24-well plate (Greiner CELLSTAR^®^, Kremsmünster, Austria). The modified BHI used in this experiment was the same as the one used in the agar plates experiments but without containing the agar. A sample was taken from the diluted culture to determine the initial bacterial concentrations by means of viability-qPCR. After 24 h of anaerobic incubation (37°C), biofilms were detached using trypsin EDTA 0.5%, followed by DNA extraction and quantification by viability-qPCR.

In addition, the inhibitory mechanism behind the effect of the L-PRF exudate was assessed in the same way as in planktonic cultures, but now in developing multi-species biofilms. Therefore, two 500-µL L-PRF exudate solutions supplemented either with HRP (80 µg/µL) or pepsin (128 µg/µL) were aerobically incubated at 37°C for 30 minutes and then added to two different wells of a 24-well plate containing the multi-species biofilm solution. These two test groups had identically final concentrations as the ones used on the agar-plate assays and planktonic cultures (40 µg/µL for HRP and 64 µg/µL for pepsin). Then, a 500-µL solution of L-PRF exudate without any enzyme supplement was considered as a positive control. Two 500-µL solutions of chlorhexidine 0.12% and 0.2% were used as additional positive controls. The negative control was obtained by the addition of 500 µL of modified BHI combined with 500 µL of *inactive* L-PRF exudate (Soltis et al., 1979). All test and control solutions were sterilized with sterile syringe filters before any anaerobic incubation.

After 24 h of anaerobic incubation (37°C), supernatants were removed. Next, treated biofilms were detached by adding 1 mL trypsin EDTA 0.05% for 5 min (37°C), centrifuged (6000 × *g* for 5 min) and re-suspended in 500 µL PBS. Afterwards, bacterial DNA was extracted from 90 µL of these samples and quantified by means of viability-qPCR in order to calculate differences in bacterial numbers, expressed as the logarithmic values of genome equivalents per millilitre [log_10_(Geq/mL)]. These differences were calculated by deducting the baseline bacterial numbers of the pre-formed biofilm for each bacterial number of the initial inoculum from the bacterial numbers obtained for each condition [(Δlog_10_(Geq/mL)].

The enzymatic blocking effect on the growth inhibition of *P. gingivalis* produced by L-PRF exudate was contrasted against a control group containing L-PRF exudate without any enzymes.

### Viability-qPCR

To extract DNA only from living bacteria, samples were first treated with propidium monoazide (PMA) (Biotium, Hayward, CA, USA) as described previously (Loozen et al., 2011; Alvarez et al., 2013). Briefly, 10 µL of PMA (final concentration of 100 µg/mL) was immediately added to 90-μL aliquots of the samples, followed by a 5-min incubation in the dark. Next, photo-induced cross-linking of PMA was achieved through a 10-min light exposure using a 400 W (500 lm) light source, placed 20 cm above the sample, while samples were kept on ice. The PMA-treated bacteria were pelleted by centrifugation at 20000 × *g* for 10 min and DNA extraction was performed using the QIAamp DNA Mini kit (Qiagen, Hilden, Germany) following the manufacturer’s instructions, but with extension of the incubation time during the bacterial lysis step with lysozyme (20 mg/mL) at 37°C to 2 h. A qPCR assay was performed with a CFX96 Real-Time System (Biorad, Hercules, CA, USA) using the Taqman 5′ nuclease assay PCR method for detection and quantification of bacterial DNA. Primers and probes were targeted against the 16 S rRNA gene for the test group and for the negative control (**Appendix 1**). Taqman reactions contained 12.5 µL Mastermix (Eurogentec, Seraing, Belgium), 4.5 µL sterile H_2_O, 1 mL of each primer and probe, and 5 µL template DNA. Assay conditions for all primer/probe sets consisted of an initial 2 min at 50°C, followed by a denaturation step at 95°C for 10 min, followed by 45 cycles of 95°C for 15 s and 60°C for 60 s. The quantification was based on a plasmid standard curve as described by Herrero and co-work*ers* (Herrero et al., 2016; **Appendix 1**).

### Statistical Analysis

Differences in the logarithmic values of genome equivalents per millilitre [Δlog_10_(Geq/mL)] between conditions were statistically analysed using a linear mixed model (pairwise comparisons of marginal linear predictions using Bonferroni’s method; adjusted across all terms) with STATA^®^ 15 and S-Plus 8.0, using the experiment number as random effect and the conditions tested as fixed effect. Mean values and standard deviations (SD) or standard errors (SE) were calculated. Corrections for simultaneous hypothesis testing was applied according to Sidak. Likelihood ratio (LR) was estimated to compare the fixed effect model against the linear mixed effect model. Outcome data distribution was graphically assessed using a Quantile-Quantile plot (Q-Q plot). For the dichotomous variable (presence or absence of bacterial growth inhibition), data were analysed using a generalized linear mixed model with experiment number as random effect and condition as fixed effect for binary data using a probit link and a continuity correction.

## Results

### Characterization of the Mechanisms Involved in the Antimicrobial Effect of L-PRF Exudate Against *P. gingivalis*

#### Agar Plate Experiments

Agar plates experiments were performed with two different agar plates because the blood agar plates may have presented a higher concentration of hemin, which could interact with peroxide (iron in hemin might detoxify peroxide) and interfere in the results.

L-PRF exudate exposed to trypsin and L-PRF exudate diluted in PBS always resulted in an inhibition of *P. gingivalis*, on both blood agar and modified BHI agar plates (**Table 1**).

**Table 1:** Antagonistic experiments on agar plates (mean ± SD).

Condition	BLOOD AGAR PLATES				MODIFIED BHI AGAR PLATES			
	Growth inhibition (n)		Average of growth inhibition area (mm^2^)	n	Growth inhibition (n)		Average of growth inhibition area (mm^2^)	n
	No	Yes			No	Yes		
*Positive control*								
CHX 0.12%	0	54	234.0 ± 27.1	54	0	69	433.2 ± 62.3	69
Undiluted L-PRF	0	54	61.2 ± 6.7	54	0	69	92.2 ± 19.2	69
L-PRF + PBS	0	54	56.4 ± 6.4	54	0	69	69.0 ± 16.9	69
*Enzymatic treatment*								
L-PRF + PBS + Pepsin	19	0	0	19	24	0	0	24
L-PRF + PBS + HRP	15	0	0	15	20	0	0	20
L-PRF + PBS + Trypsin	0	20	59.9 ± 5.5	20	0	25	76.5 ± 14.2	25
*Negative control*								
Inactive L-PRF	54	0	0	54	69	0	0	69
PBS	54	0	0	54	69	0	0	69

CHX, Chlorhexidine; L-PRF, Leucocyte- and platelet rich fibrin exudate; PBS, Phosphate-buffered saline; HRP, Horseradish peroxidase; n, number of samples.

**Figure 1:**
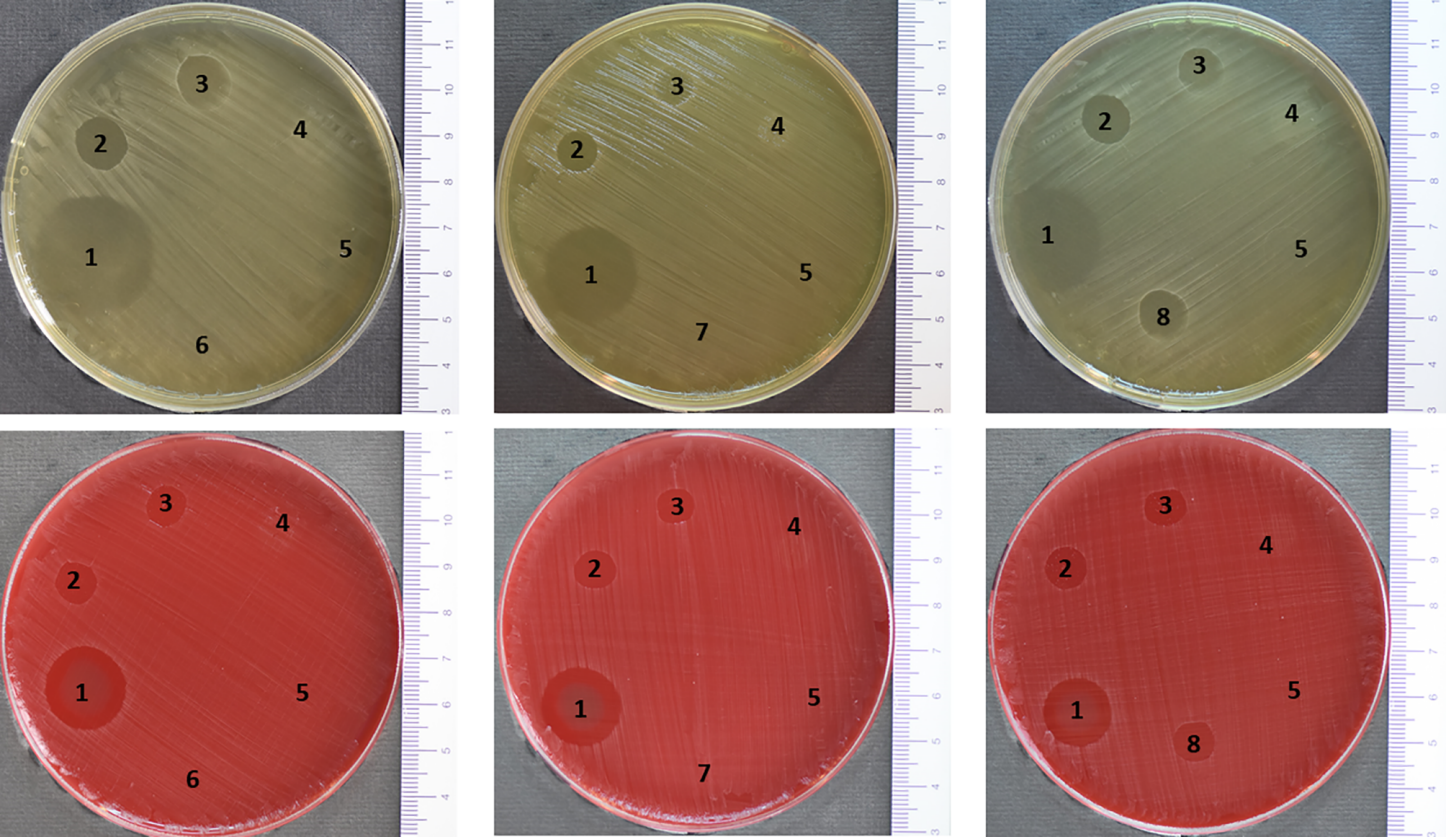
Inhibition areas presented on blood and modified BHI agar plates previously inoculated with an overnight culture of *P. gingivalis* after exposure to the following conditions: (1) CHX 0.12%, (2) Undiluted L-PRF, (3) L-PRF + PBS, (4) Inactive L-PRF, (5) PBS, (6) L-PRF + PBS + Pepsin, (7) L-PRF + PBS + HRP or (8) L-PRF + PBS + Trypsin.

Undiluted L-PRF exudate presented a larger inhibition area than L-PRF exudate exposed to trypsin in modified BHI agar (p<0.001). However, no statistically significant difference was found in blood agar plates between undiluted L-PRF exudate and trypsin-treated L-PRF exudate (p=0.962). This result may be attributed to the different composition of each medium and its possible interactions with trypsin. Undiluted L-PRF exudate presented a statistically significant larger inhibition area than L-PRF exudate diluted in PBS (p<0.001 for both, blood and modified BHI agar). Chlorhexidine conditions always presented a larger inhibition areas than the other conditions, both on blood and modified BHI agar plates (p<0.001). L-PRF exudate exposed to horseradish peroxidase (HRP) or pepsin never showed an inhibition of *P. gingivalis*. Likewise, inactive L-PRF exudate also showed no inhibition of *P. gingivalis* neither on blood agar plates, nor on modified BHI agar plates (**Table 1** and **Figure 1**). The comparisons with the statistical analysis are shown in **Appendix 2**. Normality assessment of the residual analysis showed that residuals were normally distributed after applying a log(x+1) transformation to the data (**Appendix 3**).

#### Planktonic Culture Experiments

L-PRF exudate statistically significantly decreased (p<0.001) the concentration of *P. gingivalis* compared to inactive L-PRF exudate [Δlog_10_(Geq/mL)=1.9]. This effect was less pronounced for L-PRF exudate + pepsin [Δlog_10_(Geq/mL)=1.5], L-PRF exudate + HRP [Δlog_10_(Geq/mL)=1.6] and PBS [Δlog_10_(Geq/mL)=1.2].

There were no statistically significant differences between inactive L-PRF exudate, L-PRF exudate + pepsin or L-PRF exudate + HRP (p>0.05 for all these comparisons).

The antimicrobial effect of L-PRF exudate on *P. gingivalis* was significantly lower than the effect of chlorhexidine 0.2% [Δlog_10_(Geq/mL=-0.6); p=0.008], but not different from chlorhexidine 0.12% (**Table 2** and **Appendix 4**).

**Table 2:** Comparisons of the effects of the different solutions on *P. gingivalis* in planktonic cultures.

Comparison*	Difference	*p-value*	95% CI
Inactive L-PRF *vs* L-PRF	1.915	<0.001	1.409 – 2.421
L-PRF + HRP *vs* L-PRF	1.628	<0.001	1.122 – 2.134
L-PRF + pepsin *vs* L-PRF	1.483	<0.001	0.977 – 1.989
PBS *vs* L-PRF	1.244	<0.001	0.738 – 1.750
CHX 0.12% *vs* L-PRF	0.322	0.999	-0.183 – 0.828
CHX 0.2% *vs* L-PRF	-0.590	0.008	-1.096 – -0.084
L-PRF + HRP *vs* Inactive L-PRF	-0.287	0.999	-0.793 – 0.218
L-PRF + pepsin *vs* Inactive L-PRF	-0.431	0.200	-0.937 – 0.074
PBS *vs* Inactive L-PRF	-0.670	0.001	-1.176 – -0.164
CHX 0.12% *vs* Inactive L-PRF	-1.593	<0.001	-2.099 – -1.087
CHX 0.2% *vs* Inactive L-PRF	-2.506	<0.001	-3.012 – -2.001
L-PRF + pepsin *vs* L-PRF + HRP	-0.144	0.999	-0.650 – 0.361
PBS *vs* L-PRF + HRP	-0.383	0.448	-0.889 – 0.122
CHX 0.12% *vs* L-PRF + HRP	-1.305	<0.001	-1.811– -0.799
CHX 0.2% *vs* L-PRF + HRP	-2.218	<0.001	-2.724 – -1.712
PBS *vs* L-PRF + pepsin	-0.238	0.999	-0.744 – 0.267
CHX 0.12% *vs* L-PRF + pepsin	-1.1613	<0.001	-1.667 – -0.655
CHX 0.2% *vs* L-PRF + pepsin	-2.07415	<0.001	-2.580 – -1.568
CHX 0.12% *vs* PBS	-0.922	<0.001	-1.428 – -0.416
CHX 0.2% *vs* PBS	-1.835	<0.001	-2.341 – -1.329
CHX 0.2% *vs* CHX 0.12%	-0.912	<0.001	-1.418 – -0.406

Δlog_10_(Geq/mL), differences in logarithmic values of genome equivalents per millilitre; CHX, Chlorhexidine; L-PRF, Leucocyte- and platelet rich fibrin exudate; PBS, Phosphate-buffered saline; HRP, Horseradish peroxidase; CI, Confidence interval.

^*^Bonferroni’s pairwise comparisons adjusted across all terms (p-values). Differences were calculated by deducting the Δlog_10_(Geq/mL) of the second condition from the Δlog_10_(Geq/mL) of the first condition. Standard error was 0.166 for all comparisons.

**Figure 2:**
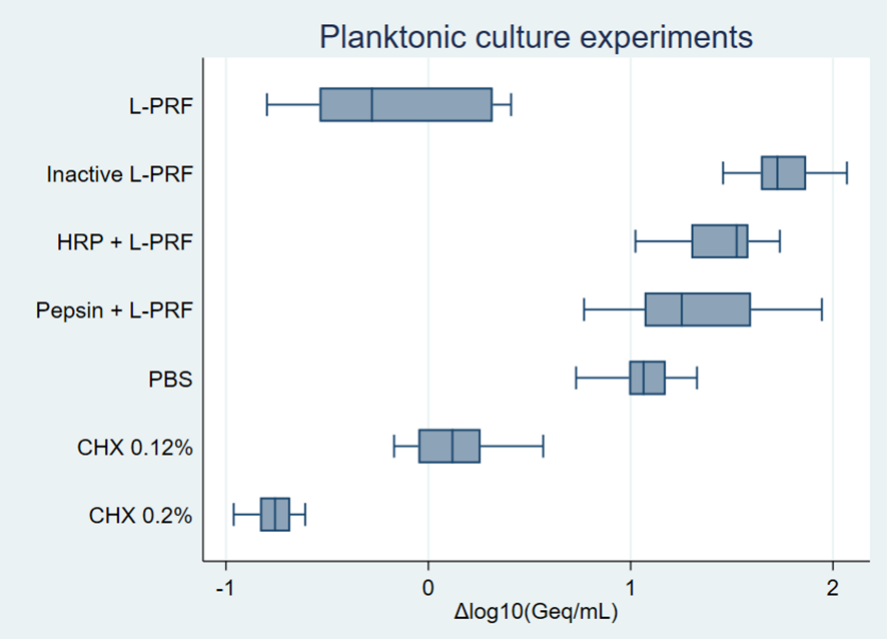
Planktonic cultures experiments *on P. gingivalis* boxplot. Negative values represent bacterial growth inhibition while positive values indicate bacterial growth. Δlog_10_(Geq/mL), Differences in logarithmic values of genome equivalents per millilitre; L-PRF, leucocyte- and platelet rich fibrin; PBS, phosphate-buffered saline; CHX, Chlorhexidine; HRP, Horseradish peroxidase.

Normality assessment of the residual analysis showed that residuals (x+1) were normally distributed (**Appendix 3**). Differences in logarithmic values of bacterial numbers among the conditions are shown in **Figure 2**.

### Effect of L-PRF Exudate Against *P. gingivalis* in a Multi-Species Biofilm

#### Pre-Formed Multi-Species Biofilm Experiments

There were no statistically significant differences between the L-PRF exudate and inactive L-PRF exudate or modified BHI in terms of decreases in *P. gingivalis* concentrations. In contrast, chlorhexidine 0.12% decreased the concentration of *P. gingivalis* in comparison to L-PRF exudate [Δlog_10_(Geq/mL)=1.4; p<0.001], inactive L-PRF exudate [Δlog_10_(Geq/mL)=1.4; p<0.001] or modified BHI [Δlog_10_(Geq/mL)=-1.4; p<0.001] (**Table 3**).

**Table 3:** Comparisons of effects of the tested solutions on *P. gingivalis* in pre-formed multi-species biofilm experiments.

Comparison^*^	Difference	*p-value*	95% CI
Inactive L-PRF *vs* L-PRF	-0.031	0.999	-0.164 – 0.103
BHI *vs* L-PRF	-0.058	0.999	-0.192 – 0.075
CHX 0.12% *vs* L-PRF	-1.429	<0.001	-1.563 – -1.295
BHI *vs* Inactive L-PRF	-0.027	0.999	-0.161 – 0.106
CHX 0.12% *vs* Inactive L-PRF	-1.399	<0.001	-1.533 – -1.265
CHX 0.12% *vs* BHI	-1.371	<0.001	-1.505 – -1.237

Δlog_10_(Geq/mL), differences in logarithmic values of genome equivalents per millilitre; CHX, Chlorhexidine; L-PRF, Leucocyte- and platelet rich fibrin exudate; BHI, Modified brain heart infusion; CI, Confidence interval.

^*^Bonferroni’s pairwise comparisons adjusted across all terms (p-values). Differences were calculated by deducting the Δlog_10_(Geq/mL) of the second condition from the Δlog_10_(Geq/mL) of the first condition. Standard error was 0.05 for all comparisons.

**Figure 3:**
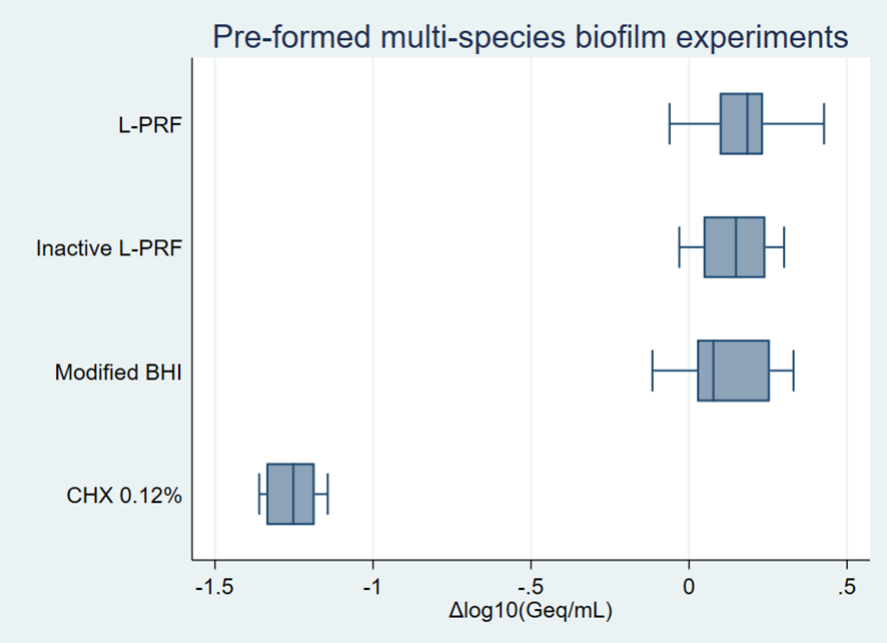
Pre-formed multi-species biofilm experiments *on P. gingivalis* boxplot. Negative values represent bacterial growth inhibition while positive values indicate bacterial growth. Δlog_10_(Geq/mL), Differences in logarithmic values of genome equivalents per millilitre; L-PRF, Leucocyte- and platelet rich fibrin; PBS, Phosphate-buffered saline; CHX, Chlorhexidine; HRP, Horseradish peroxidase.

Normality assessment of the residual analysis showed that residuals (x+1) were normally distributed (**Appendix 3**). Differences in logarithmic values of bacterial numbers among the conditions for *P. gingivalis* are shown in **Figure 3**. No effect of L-PRF exudate was found for the other bacterial species (**Appendix 5** and **6**).

#### Developing Multi-Species Biofilm Experiments

L-PRF exudate decreased the concentration of *P. gingivalis* in comparison with inactive L-PRF exudate [Δlog_10_(Geq/mL)=1.8; p<0.001], PBS [Δlog_10_(Geq/mL)=1.1; p=0.006] and L-PRF exudate exposed to HRP [Δlog_10_(Geq/mL)=1.3; p<0.001] (**Table 4**).

**Table 4:** Comparisons of the effects of the tested solutions on *P. gingivalis* in developing multi-species biofilm experiments.

Comparison^*^	Difference	*p-value*	95% CI
Inactive L-PRF *vs* L-PRF	1.782	<0.001	0.873 – 2.691
PBS *vs* L-PRF	1.081	0.006	0.173 – 1.991
L-PRF + HRP *vs* L-PRF	1.340	<0.001	0.431 – 2.248
L-PRF + pepsin *vs* L-PRF	-0.051	0.999	-0.961 – 0.859
CHX 0.12% *vs* L-PRF	0.388	0.999	-0.521 – 1.297
CHX 0.2% *vs* L-PRF	-0.845	0.099	-1.754 – 0.063
PBS *vs* Inactive L-PRF	-0.701	0.402	-1.609 – 0.208
L-PRF + HRP *vs* Inactive L-PRF	-0.442	0.999	-1.351 – 0.466
L-PRF + pepsin *vs* Inactive L-PRF	-1.833	<0.001	-2.743 – -0.922
CHX 0.12% *vs* Inactive L-PRF	-1.394	<0.001	-2.302 – -0.485
CHX 0.2% *vs* Inactive L-PRF	-2.627	<0.001	-3.536 – -1.719
L-PRF + HRP *vs* PBS	0.258	0.999	-0.650– 1.167
L-PRF + pepsin *vs* PBS	-1.132	0.003	-2.042 – -0.222
CHX 0.12% *vs* PBS	-0.693	0.429	-1.602 – 0.215
CHX 0.2% *vs* PBS	-1.927	<0.001	-2.835 – -1.018
L-PRF + pepsin *vs* L-PRF + HRP	-1.391	<0.001	-2.301 – -0.480
CHX 0.12% *vs* L-PRF + HRP	-0.951	0.031	-1.860 – -0.043
CHX 0.2% *vs* L-PRF + HRP	-2.185	<0.001	-3.094– -1.276
CHX 0.12% *vs* L-PRF + pepsin	1.782	0.999	-0.471 – 1.349
CHX 0.2% *vs* L-PRF + pepsin	1.081	0.168	-1.704 – 0.115
CHX 0.2% *vs* CHX 0.12%	1.341	0.001	-2.142 – -0.324

Δlog_10_(Geq/mL), differences in logarithmic values of genome equivalents per millilitre; CHX, Chlorhexidine; L-PRF, Leucocyte- and platelet rich fibrin exudate; PBS, Phosphate-buffered saline; HRP, Horseradish peroxidase; CI, Confidence interval.

^*^Bonferroni’s pairwise comparisons adjusted across all terms (p-values). Differences were calculated by deducting the Δlog_10_(Geq/mL) of the second condition from the Δlog_10_(Geq/mL) of the first condition. Standard error was 0.299 for all comparisons.

However, and in contrast with the planktonic cultures experiments, there were no statistically significant differences between L-PRF exudate and L-PRF exudate exposed to pepsin (p=0.999). Moreover, no statistically significant differences were found between L-PRF exudate exposed to pepsin and both chlorhexidine concentrations, 0.12% (p=0.999) or 0.2% (p=0.168).

Furthermore, there were also no statistically significant differences between L-PRF exudate and both chlorhexidine solutions, 0.12% (p=0.999) or 0.2% (p=0.099) (**Table 4**).

**Figure 4:**
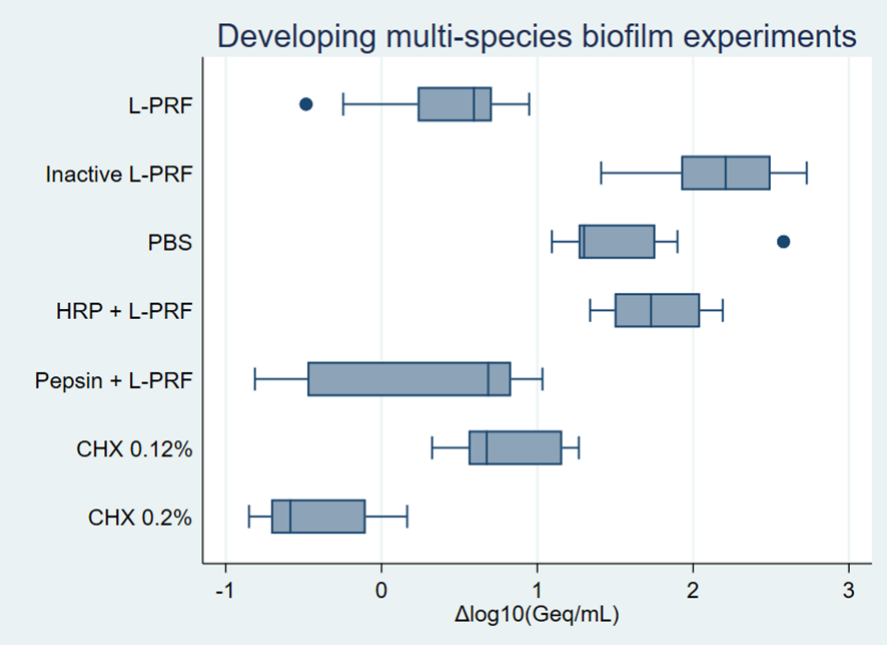
Developing multi-species biofilm experiments *on P. gingivalis* boxplot. Negative values represent bacterial growth inhibition while positive values indicate bacterial growth. Δlog_10_(Geq/mL), Differences in logarithmic values of genome equivalents per millilitre; L-PRF, Leucocyte- and platelet rich fibrin; PBS, Phosphate-buffered saline; CHX, Chlorhexidine; HRP, Horseradish peroxidase.

Normality assessment of the residual analysis showed that residuals (x+1) were normally distributed (**Appendix 3**). Differences in logarithmic values of bacterial numbers among the conditions for *P. gingivalis* are shown in **Figure 4**. No effect of L-PRF exudate was found for the other bacterial species (**Appendices 7**–**10**).

## Discussion

This study showed that L-PRF exudate caused the growth inhibition of *P. gingivalis* on agar plates, in planktonic cultures and during the development of *in vitro* multi-species biofilms. This antimicrobial effect was blocked in all models by exposing the L-PRF exudate to horseradish peroxidase. Pepsin showed similar blocking effects on L-PRF exudate, with the exception of in the developing multi-species biofilm model.

Although previous studies demonstrated the antimicrobial capability of L-PRF membranes and exudate against *P. gingivalis*, the exact mechanisms underlying this effect has not been completely revealed yet (Castro et al., 2019b). A preceding study found that PRF contained immunoglobulin G (IgG) that may provide some infection control against periodontal bacteria during the postoperative wound healing period (Yaprak et al., 2018). Another study investigating injectable PRF (i-PRF) reported that its antimicrobial and antibiofilm activity was probably related to permeability proteins, more specifically lactoferrin, defensins, heparin binding protein, cathelicidins and phospholipase A2 (Jasmine et al., 2020). The authors of this study also stated that the inhibitory and bactericidal effect of i-PRF was due to its composition of platelets, fibrin, fibronectin, thrombin, HBD-3 peptide (antimicrobial peptide), myeloperoxidase and inclusion of white blood cells (Jasmine et al., 2020). In a recent study, bacterial biofilms retrieved from the subgingival plaque of a volunteer with a relatively healthy periodontium were cultivated on a sandblasted, large grit, acid-etched titanium disk (Schuldt et al., 2020). After exposing the biofilms to L-PRF, numerous pores/perforations were found in the bacterial cell membranes and PF-4/CXCL4 kinocidins containing antimicrobial peptides were detected. The authors of this study associated the bacterial lysis produced by the platelets from L-PRF to the action of these peptides against the hydrophobic bacterial cell membranes (Schuldt et al., 2020).

However, the direct relation between those peptides and the antimicrobial capacity of the L-PRF had not been shown yet. Concerning the L-PRF exudate, no specific mechanisms of blood-derived products were known for the antimicrobial capability against *P. gingivalis*. As demonstrated herein and suggested in previous studies, an antimicrobial effect may exist in L-PRF exudate (Castro et al., 2019b). According to the findings of the present study, platelets contained in the L-PRF exudate may contribute to its antimicrobial potential by generating hydrogen peroxide and antimicrobial peptides. Some human platelet antimicrobial peptides have already been identified in the material released from human platelets, such as platelet factor 4, fibrinopeptide A, fibrinopeptide B, thymosin β-4, platelet basic protein, connective tissue activating peptide 3 and RANTES. Additionally, platelets have the capacity to generate antimicrobial oxygen metabolites including superoxide, hydrogen peroxide, and hydroxyl free radicals (Tang et al., 2002).

Considering the findings of the current study obtained for planktonic cultures and developing multi-species biofilms, the antimicrobial effect of L-PRF exudate may be qualified as bactericidal rather than bacteriostatic. These results are in contrast with the conclusions of a previous systematic review of pre-clinical evidence investigating the antimicrobial properties of platelet-rich preparations (Fabbro et al., 2016).

However, no antimicrobial effect of L-PRF exudate against *P. gingivalis* could be observed in a pre-formed multi-species biofilm in the present study. The differences found in the antimicrobial effect of L-PRF exudate against *P gingivalis* between the pre-formed and the developing multi-species biofilm may be due to the nature of the biofilm in its developing phases. It has been suggested that hydrogen peroxide plays an important role in the formation and the composition of oral biofilms (Zhu and Kreth, 2012). Some commensal species suppress the amounts of pathobionts in oral biofilms by hydrogen peroxide production, but this effect is neutralized when these biofilms persist over longer periods or become more abundant (Herrero et al., 2016). The hydrogen peroxide within the L-PRF exudate may act as an additional source, which is reflected in the decrease of *P. gingivalis* growth in developing multi-species biofilms. However, the L-PRF exudate did not influence established biofilms. The extracellular polymeric substance layer and a reduced metabolic state of the bacteria in the deeper layers may be responsible for the decreased susceptibility of the established biofilms to the L-PRF exudate, similar to what has been described for the effect of conventional antimicrobials on established biofilms (Corbin et al., 2011).

No enzymatic conditions were tested from the beginning in the developing multi-species biofilm experiments with trypsin. These conditions were previously performed in the planktonic culture experiments and trypsin did not show any blocking effect on the antimicrobial capacity of L-PRF exudate against *P. gingivalis*. Therefore, it was considered not relevant to include the enzymatic conditions with trypsin in the developing multi-species biofilm experiments.

All experiments in this study were performed with L-PRF exudate obtained from the blood of one volunteer. This methodology was chosen to decrease variability across the experiments and consequently increase the accuracy of the results. Although this may be considered as a limitation since the effect of the L-PRF exudate could be patient specific. However, a previous study could not find, from 9 volunteers, an L-PRF exudate that was not active against *P. gingivalis* (Castro et al., 2019b).

All assays in this study were performed with the same strain of *P. gingivalis* (ATCC 33277). Therefore, the findings of the present study might not be extrapolatable to other clinical strains of *P. gingivalis*.

To the best of our knowledge, this study was the first one to investigate the underlying mechanisms responsible for the antimicrobial effect of L-PRF exudate against *P. gingivalis* in different models simultaneously. Moreover, this was also the first time that the antimicrobial effect of L-PRF exudate against *P. gingivalis* was assessed on established and developing multi-species biofilms.

Within the limitations of this study, it can be concluded that L-PRF exudate may release peroxide and peptides, which may be responsible for its antimicrobial effect against *P. gingivalis*. In addition, L-PRF exudate may have an antimicrobial effect against *P. gingivalis* in developing *in vitro* multi-species biofilms. Future research is required to evaluate the effect of L-PRF on different strains of diverse bacterial species to investigate the clinical relevance of these findings.

## Data Availability Statement

The raw data supporting the conclusions of this article will be made available by the authors, without undue reservation.

## Ethics Statement

The studies involving human participants were reviewed and approved by KU Leuven ethics committee. The patients/participants provided their written informed consent to participate in this study.

## Author Contributions

Conceptualization: FR, AC, WT, and MQ. Methodology: FR, AC, and MP. Analysis: FR and CR. Writing-original draft: FR. Writing-review and editing: AC, TV, WT, and MQ. Resources: WT and MQ. Supervision: WT and MQ. All authors contributed to the article and approved the submitted version.

## Conflict of Interest

The authors declare that the research was conducted in the absence of any commercial or financial relationships that could be construed as a potential conflict of interest.

## Publisher’s Note

All claims expressed in this article are solely those of the authors and do not necessarily represent those of their affiliated organizations, or those of the publisher, the editors and the reviewers. Any product that may be evaluated in this article, or claim that may be made by its manufacturer, is not guaranteed or endorsed by the publisher.
