# Supplementary material for: Antimicrobial Mechanisms of Leucocyte- and Platelet Rich Fibrin Exudate Against Planktonic Porphyromonas gingivalis and Within Multi-Species Biofilm: A Pilot Study
Source: Front Cell Infect Microbiol. 2021 Oct 13;11:722499. doi: 10.3389/fcimb.2021.722499 (PMC8548765; doi:10.3389/fcimb.2021.722499)
Supplement: Supplementary file 1 [file DataSheet_1.pdf]

Appendix 1. TaqMan primers and probes used for the detection and quantification by viability qPCR.

**Strain**		**Primer/Probe (5’-3’)/Final concentrations**	**Length**
Aggregatibacter actinomycetemcomitans<br>16S rRNA gen	Forward<br>Reverse<br>Probe	GAA CCT TAC CTA CTC TTG ACA TCC GAA (300 nM)<br>TGC AGC ACC TGT CTC AAA GC (300 nM)<br>AGA ACT CAG AGA TGG GTT TGT GCC TTA GGG (100 nM)	80 bp
Fusobacterium nucleatum<br>*16S rRNA gen*	Forward<br>Reverse<br>Probe	GGA TTT ATT GGG CGT AAA GC (300 nM)<br>GGC ATT CCT ACA AAT ATC TAC GAA (300 nM)<br>CTC TAC ACT TGT AGT TCC G (300 nM)	162 bp
Porphyromonas gingivalis<br>*16S rRNA gen*	Forward<br>Reverse<br>Probe	GCG CTC AAC GTT CAG CC (300 nM)<br>CAC GAA TTC CGC CTG C (300 nM)<br>CAC TGA ACT CAA GCC CGG CAG TTT CAA (100 nM)	68 bp
Prevotella intermedia<br>*16S rRNA gen*	Forward<br>Reverse<br>Probe	CGG TCT GTT AAG CGT GTT GTG (300 nM)<br>CAC CAT GAA TTC CGC ATA CG (900 nM)<br>TGG CGG ACT TGA GTG CAC GC (200 nM)	99 bp
Streptococcus mutans<br>*gtfB* gen	Forward<br>Reverse<br>Probe	GCC TAC AGC TCA GAG ATG CTA TTC T (900 nM)<br>GCC ATA CAC CAC TCA TGA ATT GA (900 nM)<br>TGG AAA TGA CGG TCG CCG TTA TGA A (100 nM)	114 bp
Streptococcus sobrinus<br>*gtfT* gen	Forward<br>Reverse<br>Probe	TTC AAA GCC AAG ACC AAG CTA GT (200 nM)<br>CCA GCC TGA GAT TCA GCT TGT (200 nM)<br>CCT GCT CCA GCG ACA AAG GCA GC (250 nM)	88 bp
Actinomyces naeslundii<br>*gene encoding unknown protein*	Forward<br>Reverse<br>Probe	TCG AAA CTC AGC AAG TAG CCG (200 nM)<br>AGA GGA GGG CCA CAA AAG AAA (200 nM)<br>GGG TAC TCT AGT CCA AAC TGG CGG ATA GCG (100 nM)	96 bp
Streptococcus gordonii<br>*gtfG gen*	Forward<br>Reverse<br>Probe	CGG ATG ATG CTA ATC AAG TGA CC (400 nM)<br>GTT AGC TGT TGG ATT GGT TGC C (400 nM)<br>AGA ACA GTC CGC TGT TCA GAG CAA (100 nM)	177 bp
Actinomyces viscosus<br>*16S rRNA gen*	Forward<br>Reverse<br>Probe	GTG AAG GAG CCA GCT TGC TGG TTC TG (200 nM)<br>CGG AAC AAA CCT TTC CCA GGC (200 nM)<br>ATG AGT GGC GAA CGG GTG AGT AAC (125 nM)	155 bp
Streptococcus salivarius<br>*Dextranase gene*	Forward<br>Reverse<br>Probe	AAC GTT GAC CTT ACG CTA GC (400 nM)<br>ACC GTA ACG TGG GAA AAC TG (400 nM)<br>GTA GCG TCA GAG TGG TTG AC (100 nM)	192 bp
Streptococcus oralis<br>*gtfR gen*	Forward<br>Reverse<br>Probe	ACC AGC AGA TAC GAA AGA AGC AT (400 nM)<br>AGG TTC GGG CAA GCG ATC TTT CT (400 nM)<br>AAG GCT GCT GTT GCT GAA GAA GT (100 nM)	229 bp
Streptococcus mitis<br>*16S rRNA gen*	Forward<br>Reverse<br>Probe	GGC TCG TAG TCT GGA GAT GG (600 nM)<br>TAG GTC GTC GTC CCA AGG AA (600 nM)<br>CGA AGA GCA CCA ATA GCA CCT CCC (140 nM)	133 bp
Streptococcus sanguinis<br>*gtfP* gen	Forward<br>Reverse<br>Probe	CAA AAT TGT TGC AAA TCC AAA GG (600 nM)<br>GCT ATC GCT CCC TGT CTT TGA (600 nM)<br>AAA GAA AGA TCG CTT GCC AGA ACC GG (100 nM)	75 bp
Veillonella parvula<br>*16S rRNA* gen	Forward<br>Reverse<br>Probe	GAC GAA AGT CTG ACG GAG CA (200 nM)<br>TGC CAC CTA CGT ATT ACC GC (200 nM)<br>AGC TCT GTT AAT CGG GAC GAA AGG C (125 nM)	171 bp

Appendix 2. Antagonistic experiments on agar plates.

Appendix 2.1. Blood agar plate experiments:

Continous variable analysis (bacterial growth inhibition area in mm2):

Comparison	Difference*	P-value
Chlorhexidine 0.12% - L-PRF+PBS	1.409	<0.001
Chlorhexidine 0.12%-Trypsin + L-PRF	1.345	<0.001
Chlorhexidine 0.12%-Inactive L-PRF	1.327	<0.001
L-PRF + PBS - Trypsin + L-PRF	-0.063	0.055
L-PRF+PBS-Undiluted L-PRF	-0.081	<0.001
Trypsin + L-PRF-Undiluted L-PRF	-0.017	0.962
Chlorhexidine 0.12%-0**	5.450	<0.001
L-PRF+PBS-0**	4.041	<0.001
Trypsin + L-PRF-0**	4.104	<0.001
Undiluted L-PRF-0**	4.122	<0.001

Note: Values were log-transformed before analysis. The numbers in the Difference column are the differences as given by the statistical model, after transformation of the data. Therefore, they are not the real differences between the measured values on the agar plates.

* Differences as given by the statistical model, after transformation of the data.

****** Difference between the first-named group and the value 0.

Dichotomous variable analysis (presence or absence of bacterial growth inhibition):

Comparison	P-value
Chlorhexidine 0.12%-Inactive L-PRF	<0.001
Chlorhexidine 0.12%-L-PRF+PBS	0.999
Chlorhexidine 0.12%-PBS	<0.001
Chlorhexidine 0.12%-Pepsin + L-PRF	<0.001
Chlorhexidine 0.12%-Peroxidase + L-PRF	<0.001
Chlorhexidine 0.12%-Trypsin + L-PRF	0.996
Chlorhexidine 0.12%-Undiluted L-PRF	0.999
Inactive L-PRF-L-PRF+PBS	<0.001
Inactive L-PRF-PBS	0.999
Inactive L-PRF-Pepsin + L-PRF	0.995
Inactive L-PRF-Peroxidase + L-PRF	0.987
Inactive L-PRF-Trypsin + L-PRF	<0.001
Inactive L-PRF-Undiluted L-PRF	<0.001
L-PRF+PBS-PBS	<0.001
L-PRF+PBS-Pepsin + L-PRF	<0.001
L-PRF+PBS-Peroxidase + L-PRF	<0.001
L-PRF+PBS-Trypsin + L-PRF	0.996
L-PRF+PBS-Undiluted L-PRF	0.999
PBS-Pepsin + L-PRF	0.995
PBS-Peroxidase + L-PRF	0.987
PBS-Trypsin + L-PRF	<0.001
PBS-Undiluted L-PRF	<0.001
Pepsin + L-PRF-Peroxidase + L-PRF	0.999

Note: This table shows the comparison in terms of presence or absence of bacterial growth inhibition between the different conditions (dichotomous variable analysis).

Appendix 2.2. Modified BHI agar plate experiments:

Continous variable analysis (bacterial growth inhibition area in mm2):

Comparison	Difference*	P-value
Chlorhexidine 0.12% - L-PRF+PBS	1.842	<0.001
Chlorhexidine 0.12% - Trypsin + L-PRF	1.724	<0.001
Chlorhexidine 0.12% - Undiluted L-PRF	1.549	<0.001
L-PRF+PBS - L-PRF + Trypsin	-0.118	0.047
L-PRF+PBS - Undiluted L-PRF	-0.293	<0.001
Trypsin + L-PRF - Undiluted L-PRF	-0.175	<0.001
Chlorhexidine 0.12% - 0**	6.060	<0.001
L-PRF+PBS - 0**	4.217	<0.001
Trypsin + L-PRF - 0**	4.336	<0.001
Undiluted L-PRF - 0**	4.511	<0.001

Note: Values were log-transformed before analysis. The numbers in the Difference column are the differences as given by the statistical model, after transformation of the data. herefore, they are not the real differences between the measured values on the agar plates.

* Differences as given by the statistical model, after transformation of the data.

****** Difference between the first-named group and the value 0.

Dichotomous variable analysis (presence or absence of bacterial growth inhibition):

Comparison	P-value
Chlorhexidine 0.12%-Inactive L-PRF	<0.001
Chlorhexidine 0.12%-Diluted L-PRF	0.999
Chlorhexidine 0.12%-PBS	<0.001
Chlorhexidine 0.12%-Pepsin + L-PRF	<0.001
Chlorhexidine 0.12%-Peroxidase + L-PRF	<0.001
Chlorhexidine 0.12%-Trypsin + L-PRF	0.996
Chlorhexidine 0.12%-Undiluted L-PRF	0.999
Inactive L-PRF - L-PRF+PBS	<0.001
Inactive L-PRF-PBS	0.999
Inactive L-PRF-Pepsin + L-PRF	0.995
Inactive L-PRF-Peroxidase + L-PRF	0.987
Inactive L-PRF-Trypsin + L-PRF	<0.001
Inactive L-PRF-Undiluted L-PRF	<0.001
L-PRF+PBS-PBS	<0.001
L-PRF+PBS-Pepsin + L-PRF	<0.001
L-PRF+PBS-Peroxidase + L-PRF	<0.001
L-PRF+PBS-Trypsin + L-PRF	0.996
L-PRF+PBS-Undiluted L-PRF	0.999
PBS-Pepsin + L-PRF	0.995
PBS-Peroxidase + L-PRF	0.987
PBS-Trypsin + L-PRF	<0.001
PBS-Undiluted L-PRF	<0.001
Pepsin + L-PRF-Peroxidase + L-PRF	0.999

Note: This table shows the comparison in terms of presence or absence of bacterial growth inhibition between the different conditions (dichotomous variable analysis).

Appendix 3. Q-Q plots

Modified BHI agar plates experiments Q-Q plots

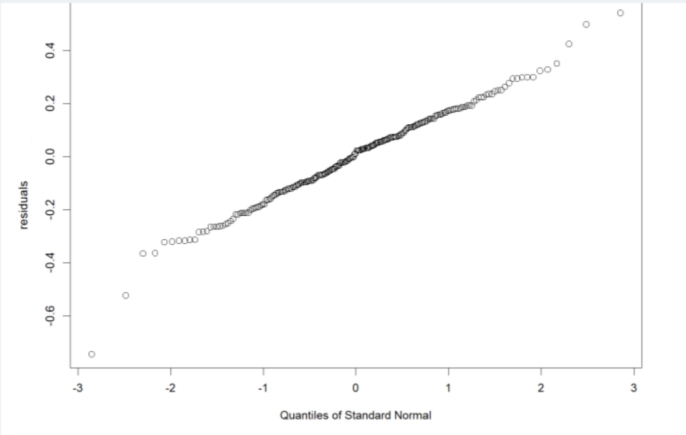

Planktonic cultures experiments Q-Q plot

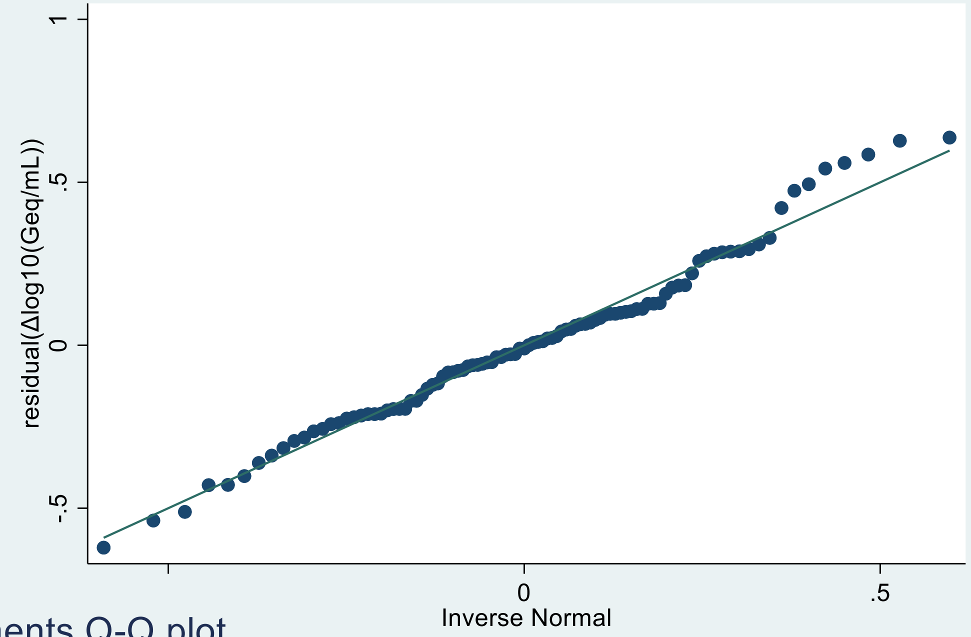

Blood agar plates experiments Q-Q plots

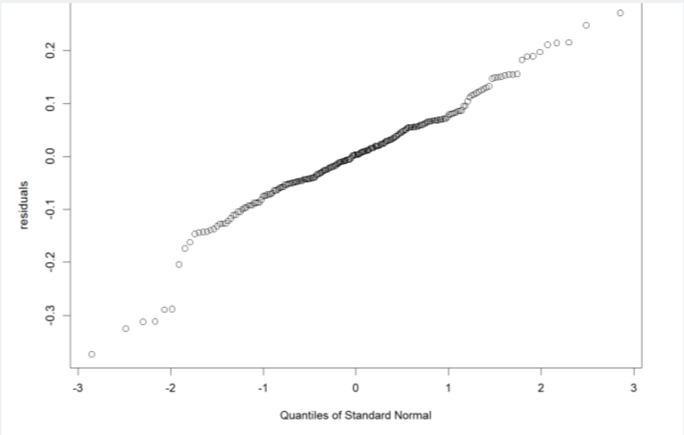

Developing multis-pecies biofilm experiments Q-Q plot

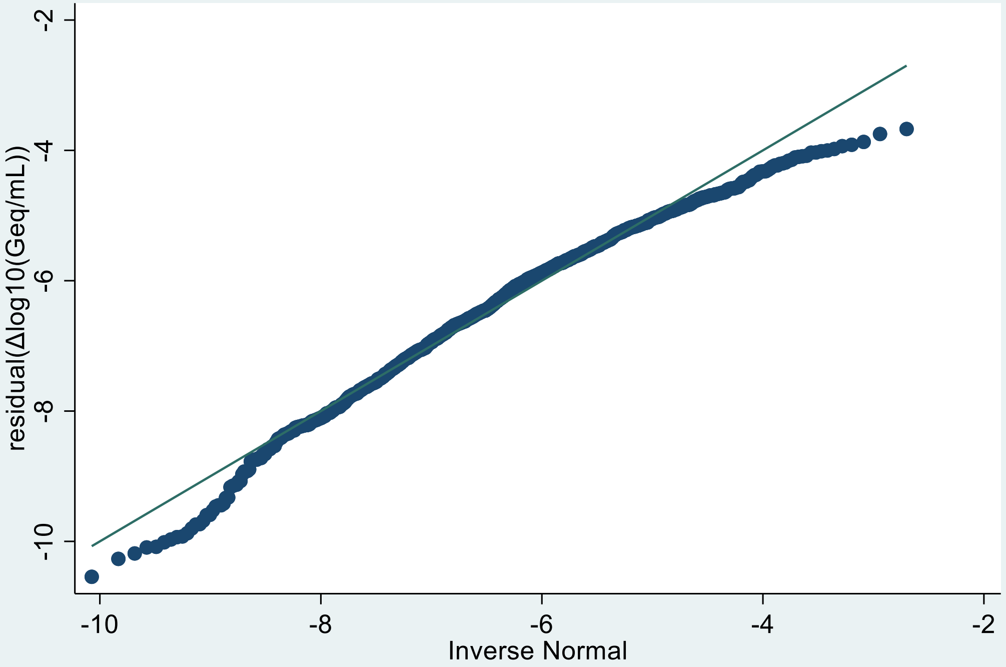

Pre-formed multi-species biofilm experiments Q-Q plot

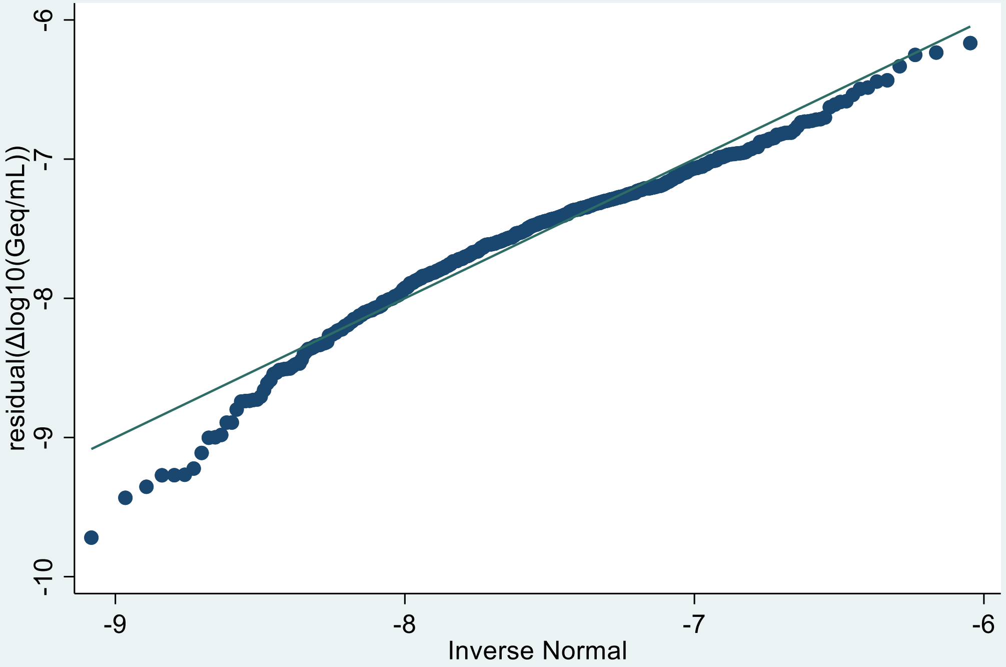

Appendix 4. Effect of the different solutions on *P. gingivalis* in planktonic culture experiments (log_{10}(Geq/mL)).

	**Mean**	**Standard deviation**	**Number of data**
**L-PRF**	6.935	0.993	14
**Inactive L-PRF**	8.834	0.700	14
**Peroxidase + L-PRF**	8.549	0.746	14
**Pepsin + L-PRF**	8.404	0.945	14
**CHX 0.12%**	7.244	0.769	14
**CHX 0.2%**	6.331	0.623	14
**PBS**	8.153	0.594	14

CHX: Chlorhexidine; L-PRF: Leucocyte- and platelet rich fibrin exudate; PBS: phosphate-buffered saline; HRP: horseradish peroxidase; SD: standard deviation.

Appendix 5. Comparisons of effects of the tested solutions on the other bacterial species in pre-formed multi-species biofilm experiments (Δlog_{10}(Geq/mL)).

Mean[a]<br>*(p-value)*<br>*SE*	**Inactive L-PRF vs L-PRF**	**Modified BHI vs L-PRF**	**CHX 0.12% vs L-PRF**	**Modified BHI vs Inactive L-PRF**	**CHX 0.12% vs Inactive L-PRF**	**CHX 0.12% vs modified BHI**
Pathogen species						
S. sobrinus	0.1<br>(1.0)<br>0.2	0.2<br>(1.0)<br>0.2	-0.6<br>(0.01)<br>0.2	0.2<br>(1.0)<br>0.2	-0.7<br>(0.01)<br>0.2	-0.9<br>(<0.001)<br>0.2
F. nucleatum	-0.1<br>(1.0)<br>0.3	-0.2<br>(1.0)<br>0.3	-2.9<br>(<0.001)<br>0.3	-0.9<br>(1.0)<br>0.3	-2.8<br>(<0.001)<br>0.3	-2.7<br>(1.0)<br>0.3
P. intermedia	-0.1<br>(1.0)<br>0.2	-0.9<br>(<0.001)<br>0.2	-2.1<br>(<0.001)<br>0.2	-0.7<br>(0.001)<br>0.2	-1.9<br>(<0.001)<br>0.2	-0.2<br>(<0.001)<br>0.2
S. mutans	-0.9<br>(<0.001)<br>0.1	-0.1<br>(0.9)<br>0.1	0.1<br>(1.0)<br>0.1	0.3<br>(0.04)<br>0.1	-0.5<br>(<0.001)<br>0.1	-0.9<br>(<0.001)<br>0.1
A. Actinomycetem-comitans	-0.2<br>(1.0)<br>0.2	-0.1<br>(1.0)<br>0.2	-0.6<br>(0.06)<br>0.2	0.1<br>(1.0)<br>0.2	-0.4<br>(0.5)<br>0.2	-0.6<br>(0.1)<br>0.2
Commensal species						
S. gordonii	-0.1<br>(<0.001)<br>0.1	-0.3<br>(0.2)<br>0.1	-2.2<br>(<0.001)<br>0.1	-0.2<br>(0.9)<br>0.1	-2.1<br>(<0.001)<br>0.1	-1.9<br>(<0.001)<br>0.1
S. mitis	-0.2<br>(1.0)<br>0.4	0.1<br>(1.0)<br>0.4	-1.1<br>(0.09)<br>0.4	0.2<br>(1.0)<br>0.4	-0.8<br>(0.3)<br>0.4	-1.1<br>(0.06)<br>0.4
A. naeslundii	0.1<br>(0.7)<br>0.2	0.4<br>(0.1)<br>0.2	0.1<br>(0.9)<br>0.2	-0.1<br>(0.6)<br>0.2	-1.4<br>(<0.001)<br>0.2	-1.6<br>(<0.001)<br>0.2
A. viscosus	-0.1<br>(1.0)<br>0.4	-0.1<br>(1.0)<br>0.4	-2.1<br>(<0.001)<br>0.4	-0.1<br>(1.0)<br>0.4	-2.1<br>(<0.001)<br>0.4	-1.9<br>(<0.001)<br>0.4
S. oralis	0.2<br>(0.8)<br>0.1	-0.6<br>(<0.001)<br>0.1	-1.2<br>(<0.001)<br>0.2	-0.8<br>(<0.001)<br>0.1	-1.4<br>(<0.001)<br>0.2	-0.6<br>(0.007)<br>0.2
S. salivarius	-0.1<br>(1.0)<br>0.1	-0.1<br>(1.0)<br>0.1	-1.1<br>(<0.001)<br>0.2	-0.1<br>(1.0)<br>0.1	-1.1<br>(<0.001)<br>0.2	-1.1<br>(<0.001)<br>0.2
S. sanguinis	-0.1<br>(0.8)<br>0.1	-0.1<br>(1.0)<br>0.1	-0.1<br>(1.0)<br>0.1	0.1<br>(1.0)<br>0.1	0.1<br>(1.0)<br>0.1	-0.1<br>(1.0)<br>0.1
V. parvula	-0.1<br>(1.0)<br>0.2	-0.1<br>(1.0)<br>0.2	-1.6<br>(<0.001)<br>0.2	-0.1<br>(1.0)<br>0.2	-1.5<br>(<0.001)<br>0.2	-1.5<br>(<0.001)<br>0.2

[a] Pairwise comparisons of marginal linear predictions using Bonferroni's method adjusted across all terms (*p*-values). Differences were calculated by deducting the Δlog(Geq/ml) of the second condition from the Δlog(Geq/ml) of the first one.

CHX: Chlorhexidine; L-PRF: Leucocyte- and platelet rich fibrin exudate; PBS: phosphate-buffered saline; HRP: horseradish peroxidase; vs: versus; SE: standard error.

Appendix 6. Effect of the tested solutions on the other bacterial species in pre-formed multi-species biofilm experiments (log_{10}(Geq/mL)).

Mean (SD) n	**Inactive L-PRF**	**L-PRF**	**CHX 0.12%**	**Modified BHI**
Pathogen species				
S. sobrinus	8.763 (0.736) 15	8.745 (0.732) 15	7.967 (0.734) 15	8.873 (0.563) 15
F. nucleatum	9.432 (0.692) 15	9.574 (0.779) 15	6.291 (0.283) 15	9.069 (0.718) 15
P. intermedia	7.854 (0.381) 15	8.019 (0.476) 15	5.639 (0.2394) 15	6.827 (0.594) 15
S. mutans	5.482 (1.228) 15	5.570 (1.046) 15	4.577 (0.728) 15	5.700 (0.917) 15
A. Actinomycetem-comitans	7.463 (1.023) 15	7.624 (0.885) 15	6.761 (0.652) 15	7.528 (0.820) 15
Commensal species				
S. gordonii	9.152 (0.977) 15	9.212 (0.929) 15	7.161 (1.255) 15	8.882 (0.892) 15
S. mitis	6.012 (0.283) 15	6.166 (0. 315) 15	5.006 (0.377) 15	6.136 (0.264) 15
A. naeslundii	7.056 (1.015) 15	7.161 (0.953) 15	6.677 (1.002) 15	7.043 (0.760) 15
A. viscosus	7.352 (0.399) 15	7.404 (0.415) 15	5.238 (0.442) 15	7.162 (0.292) 15
S. oralis	5.606 (0.420) 15	5.432 (0.598) 15	4.232 (0.267) 15	4.907 (0.398) 15
S. salivarius	5.265 (0.976) 11	5.188 (1.075) 12	3.417 (-) 1	4.873 (0.906) 12
S. sanguinis	5.617 (0.411) 15	5.740 (0.332) 15	5.429 (0.155) 15	5.637 (0.237) 15
V. parvula	10.026 (0.386) 15	10.076 (0.347) 15	8.264 (0.357) 15	9.922 (0.265) 15

CHX: Chlorhexidine; L-PRF: Leucocyte- and platelet rich fibrin exudate; PBS: phosphate-buffered saline; HRP: horseradish peroxidase; SD: standard deviation; n: number of data.

Appendix 7. Comparisons of the effects of the tested solutions on pathogen species in developing multi-species biofilm experiments (Δlog_{10}(Geq/mL)).

Mean[a]<br>*(p-value)*<br>*SE*	***S. sobrinus***	***F. nucleatum***	***P. intermedia***	***S. mutans***	***A. Actinomycetem-comitans***
**Inactive L-PRF vs L-PRF**	0.17<br>(1.0)<br>0.3	0.2<br>(1.0)<br>0.3	-0.4<br>(1.0)<br>0.3	-0.1<br>(1.0)<br>0.4	0.1<br>(1.0)<br>0.6
**PBS vs L-PRF**	1.55<br>(<0.001)<br>0.3	-1.1<br>(0.08)<br>0.3	-2.9<br>(<0.001)<br>0.3	0.2<br>(1.0)<br>0.4	-0.1<br>(1.0)<br>0.6
**Peroxidase + L-PRF vs L-PRF**	-0.1<br>(1.0)<br>0.3	-0.1<br>(1.0)<br>0.3	-0.4<br>(1.0)<br>0.3	-0.1<br>(1.0)<br>0.4	-0.2<br>(1.0)<br>0.6
**Pepsin + L-PRF vs L-PRF**	0.1<br>(1.0)<br>0.3	-2.4<br>(<0.001)<br>0.3	-1.5<br>(<0.001)<br>0.3	-0.3<br>(1.0)<br>0.4	-0.7<br>(1.0)<br>0.6
**CHX 0.12% vs L-PRF**	-1.2<br>(0.001)<br>0.3	-1.8<br>(<0.001)<br>0.3	-2.7<br>(<0.001)<br>0.3	-0.8<br>(1.0)<br>0.4	0.3<br>(1.0)<br>0.6
**CHX 0.2% vs L-PRF**	-1.4<br>(<0.001)<br>0.3	-3.1<br>(<0.001)<br>0.3	-3.8<br>(<0.001)<br>0.3	-0.8<br>(1.0)<br>0.4	-0.7<br>(1.0)<br>0.6
**PBS vs Inactive L-PRF**	1.3<br>(<0.001)<br>0.3	-1.1<br>(0.06)<br>0.3	-2.5<br>(<0.001)<br>0.3	0.3<br>(1.0)<br>0.4	-0.2<br>(1.0)<br>0.6
**Peroxidase + L-PRF vs Inactive L-PRF**	-0.3<br>(1.0)<br>0.3	-0.1<br>(1.0)<br>0.3	-0.1<br>(1.0)<br>0.3	0.1<br>(1.0)<br>0.4	-0.4<br>(1.0)<br>0.6
**Pepsin + L-PRF vs Inactive L-PRF**	0.1<br>(1.0)<br>0.3	-2.4<br>(<0.001)<br>0.3	-1.1<br>(0.004)<br>0.3	-0.1<br>(1.0)<br>0.4	-0.9<br>(1.0)<br>0.6
**CHX 0.12% vs Inactive L-PRF**	-1.3<br>(<0.001)<br>0.3	-1.8<br>(<0.001)<br>0.3	-2.3<br>(<0.001)<br>0.3	-0.7<br>(1.0)<br>0.4	0.1<br>(1.0)<br>0.6
**CHX 0.2% vs Inactive L-PRF**	-1.6<br>(<0.001)<br>0.3	-3.1<br>(<0.001)<br>0.3	-3.3<br>(<0.001)<br>0.3	-0.6<br>(1.0)<br>0.4	-0.9<br>(1.0)<br>0.6
**Peroxidase + L-PRF vs PBS**	-1.7<br>(<0.001)<br>0.3	1.1<br>(0.09)<br>0.3	2.5<br>(<0.001)<br>0.3	-0.3<br>(1.0)<br>0.4	-0.1<br>(1.0)<br>0.6
**Pepsin + L-PRF vs PBS**	-1.3<br>(<0.001)<br>0.3	-1.4<br>(0.002)<br>0.3	1.4<br>(<0.001)<br>0.3	-0.5<br>(1.0)<br>0.4	-0.7<br>(1.0)<br>0.6
**CHX 0.12% vs PBS**	-2.7<br>(<0.001)<br>0.3	-0.8<br>(0.4)<br>0.3	0.1<br>(1.0)<br>0.3	-1.1<br>(0.3)<br>0.4	0.4<br>(1.0)<br>0.6

**CHX 0.2% vs PBS**	-2.9 (<0.001) 0.293	-2.1 (<0.001) 0.3	-0.8 (0.05) 0.3	-1.1 (0.4) 0.4	-0.6 (1.0) 0.6
**Pepsin + L-PRF vs Peroxidase + L-PRF**	0.3 (1.0) 0.3	-2.4 (<0.001) 0.3	-1.1 (0.002) 0.3	-1.9 (1.0) 0.4	-0.5 (1.0) 0.6
**CHX 0.12% vs Peroxidase + L-PRF**	-1.1 (0.01) 0.3	-1.8 (<0.001) 0.3	-2.3 (<0.001) 0.3	-0.7 (1.0) 0.4	0.6 (1.0) 0.6
**CHX 0.2% vs Peroxidase + L-PRF**	-1.2 (<0.001) 0.3	-3.1 (<0.001) 0.3	-3.4 (<0.001) 0.3	-0.6 (1.0) 0.4	-0.5 (1.0) 0.6
**CHX 0.12% vs Pepsin + L-PRF**	-1.3 (<0.001) 0.3	0.5 (1.0) 0.3	-1.2 (0.001) 0.3	-0.5 (1.0) 0.4	1.1 (1.0) 0.6
**CHX 0.2% vs Pepsin + L-PRF**	-1.6 (<0.001) 0.3	-0.6 (1.0) 0.3	-2.2 (<0.001) 0.3	-0.4 (1.0) 0.4	-0.3 (1.0) 0.6
**CHX 0.2% vs CHX 0.12%**	-0.2 (<0.001) 0.3	-1.2 (0.01) 0.3	-1.7 (0.005) 0.3	-0.1 (1.0) 0.4	-1.1 (1.0) 0.6

[a] Pairwise comparisons of marginal linear predictions using Bonferroni's method adjusted across all terms (*p*-values). Differences were calculated by deducting the Δlog(Geq/ml) of the second condition from the Δlog(Geq/ml) of the first one.

CHX: Chlorhexidine; L-PRF: Leucocyte- and platelet rich fibrin exudate; PBS: phosphate-buffered saline; HRP: horseradish peroxidase; vs: versus; SE: standard error.

Appendix 8. Comparisons of the effects of the tested solutions on commensal species in developing multi-species biofilm experiments (Δ$\log_{10}$(Geq/mL)).

Mean[a] (p-value) Std error	***S. gordonii***	***S. mitis***	***A. naeslundii***	***A. viscosus***	***S. oralis***	***S. salivarius***	***S. sanguinis***	***V. parvula***
**Inactive L-PRF vs L-PRF**	-0.1 (1.0) 0.3	-0.1 (1.0) 0.5	0.1 (1.0) 0.2	0.2 (1.0) 0.2	-0.3 (1.0) 0.7	-0.1 (1.0) 0.3	-0.1 (1.0) 0.2	-0.1 (1.0) 0.1
**PBS vs L-PRF**	-0.4 (1.0) 0.3	0.1 (1.0) 0.5	0.4 (1.0) 0.2	0.1 (1.0) 0.2	-1.1 (1.0) 0.7	-0.2 (1.0) 0.3	-0.1 (1.0) 0.2	-0.1 (1.0) 0.1
**Peroxidase + L-PRF vs L-PRF**	-0.1 (1.0) 0.3	0.3 (1.0) 0.5	0.1 (1.0) 0.2	-0.1 (1.0) 0.2	-0.5 (1.0) 0.7	-0.1 (1.0) 0.3	-0.2 (1.0) 0.2	-0.2 (1.0) 0.1
**Pepsin + L-PRF vs L-PRF**	-0.7 (1.0) 0.3	-0.3 (1.0) 0.5	-0.1 (1.0) 0.2	-0.5 (0.6) 0.2	-1.3 (1.0) 0.7	-0.7 (1.0) 0.4	-0.4 (1.0) 0.2	-0.1 (<0.001) 0.1
**CHX 0.12% vs L-PRF**	-2.9 (<0.001) 0.3	-0.1 (1.0) 0.5	-1.4 (<0.001) 0.2	-2.2 (<0.001) 0.2	-1.5 (0.6) 0.7	-0.6 (1.0) 0.3	-1.2 (<0.001) 0.2	-1.9 (<0.001) 0.1
**CHX 0.2% vs L-PRF**	-3.3 (<0.001) 0.3	-0.3 (1.0) 0.5	-1.6 (<0.001) 0.2	-2.5 (<0.001) 0.2	-2.3 (0.02) 0.7	-0.5 (1.0) 0.5	-1.1 (<0.001) 0.2	-2.8 (<0.001) 0.1
**PBS vs Inactive L-PRF**	-0.3 (1.0) 0.3	0.1 (1.0) 0.5	0.3 (1.0) 0.2	-0.1 (1.0) 0.2	-0.8 (1.0) 0.7	-0.1 (1.0) 0.3	-0.1 (1.0) 0.2	-0.1 (1.0) 0.1
**Peroxidase + L-PRF vs Inactive L-PRF**	-0.1 (1.0) 0.3	0.4 (1.0) 0.5	-0.1 (1.0) 0.2	-0.3 (1.0) 0.2	-0.2 (1.0) 0.7	-0.1 (1.0) 0.3	-0.1 (1.0) 0.2	-0.1 (1.0) 0.1
**Pepsin + L-PRF vs Inactive L-PRF**	-0.6 (1.0) 0.3	-0.2 (1.0) 0.5	-0.1 (1.0) 0.2	-0.8 (0.02) 0.2	-1.1 (1.0) 0.7	-0.6 (1.0) 0.3	-0.3 (1.0) 0.2	-0.9 (<0.001) 0.1
**CHX 0.12% vs Inactive L-PRF**	-2.8 (<0.001) 0.3	-0.1 (1.0) 0.5	-1.4 (<0.001) 0.2	-2.4 (<0.001) 0.2	-1.2 (1.0) 0.7	-0.5 (1.0) 0.3	-1.1 (<0.001) 0.2	-1.8 (<0.001) 0.1
**CHX 0.2% vs Inactive L-PRF**	-3.2 (<0.001) 0.3	-0.3 (1.0) 0.5	-1.7 (<0.001) 0.2	-2.8 (<0.001) 0.2	-2.1 (0.1) 0.7	-0.4 (1.0) 0.5	-0.9 (0.002) 0.2	-2.8 (<0.001) 0.1
**Peroxidase + L-PRF vs PBS**	0.2 (1.0) 0.3	0.3 (1.0) 0.5	-0.3 (1.0) 0.2	-0.2 (1.0) 0.2	0.6 (1.0) 0.7	-0.1 (1.0) 0.3	-0.1 (1.0) 0.2	-0.1 (1.0) 0.1
**Pepsin + L-PRF vs PBS**	-0.2 (1.0) 0.3	-0.4 (1.0) 0.5	-0.5 (0.8) 0.2	-0.7 (0.08) 0.2	-1.7 (1.0) 0.7	-0.5 (1.0) 0.3	-0.3 (1.0) 0.2	-0.9 (<0.001) 0.1
**CHX 0.12% vs PBS**	-2.4 (<0.001) 0.3	-0.1 (1.0) 0.5	-1.8 (<0.001) 0.2	-2.4 (<0.001) 0.2	-0.3 (1.0) 0.7	-0.4 (1.0) 0.3	-1.1 (<0.001) 0.2	-1.8 (<0.001) 0.1

**CHX 0.2% vs PBS**	-2.9 (<0.001) 0.3	-0.4 (1.0) 0.5	-2.1 (<0.001) 0.2	-2.7 (<0.001) 0.2	-1.1 (1.0) 0.7	-0.3 (1.0) 0.5	-0.9 (0.002) 0.2	-2.8 (<0.001) 0.1
**Pepsin + L-PRF vs Peroxidase + L-PRF**	-0.5 (1.0) 0.3	-0.7 (1.0) 0.5	-0.1 (1.0) 0.2	-0.4 (1.0) 0.2	-0.8 (1.0) 0.7	-0.5 (1.0) 0.3	-0.1 (1.0) 0.2	-0.7 (<0.001) 0.1
**CHX 0.12% vs Peroxidase + L-PRF**	-2.7 (<0.001) 0.3	-0.4 (1.0) 0.5	-1.4 (<0.001) 0.2	-2.1 (<0.001) 0.2	-1.1 (1.0) 0.7	-0.5 (1.0) 0.3	-0.9 (<0.001) 0.2	-1.7 (<0.001) 0.1
**CHX 0.2% vs Peroxidase + L-PRF**	-3.1 (<0.001) 0.3	-0.7 (1.0) 0.5	-1.6 (<0.001) 0.2	-2.4 (<0.001) 0.2	-1.8 (0.2) 0.7	-0.3 (1.0) 0.5	-0.7 (0.02) 0.2	-2.6 (<0.001) 0.1
**CHX 0.12% vs Pepsin + L-PRF**	-2.2 (<0.001) 0.3	0.2 (1.0) 0.5	-1.2 (<0.001) 0.2	-1.6 (<0.001) 0.2	-0.2 (1.0) 0.7	0.1 (1.0) 0.4	-0.8 (0.007) 0.2	-0.9 (<0.001) 0.1
**CHX 0.2% vs Pepsin + L-PRF**	-2.6 (<0.001) 0.3	-0.1 (1.0) 0.5	-1.5 (<0.001) 0.2	-2.0 (<0.001) 0.2	-0.9 (1.0) 0.7	0.1 (1.0) 0.3	-0.5 (0.2) 0.2	-1.8 (<0.001) 0.1
**CHX 0.2% vs CHX 0.12%**	-0.4 (1.0) 0.3	-0.2 (1.0) 0.5	-0.2 (1.0) 0.2	-0.3 (1.0) 0.2	-0.7 (1.0) 0.7	0.1 (1.0) 0.5	0.2 (1.0) 0.2	-0.1 (<0.001) 0.1

[a] Pairwise comparisons of marginal linear predictions using Bonferroni's method adjusted across all terms (*p*-values). Differences were calculated by deducting the Δlog(Geq/ml) of the second condition from the Δlog(Geq/ml) of the first one.

CHX: Chlorhexidine; L-PRF: Leucocyte- and platelet rich fibrin exudate; PBS: phosphate-buffered saline; HRP: horseradish peroxidase; vs: versus; SE: standard error.

Appendix 9. Effects of the tested solutions on pathogen species in developing multi-species biofilm experiments (log_{10}(Geq/mL)).

Mean (SD) n	***S. sobrinus***	***F. nucleatum***	***P. intermedia***	***S. mutans***	***A. Actinomycetem-comitans***
**L-PRF**	7.485 (0.585) 14	9.703 (1.066) 14	9.696 (0.400) 14	5.471 (1.015) 14	7.653 (0.874) 14
**Inactive L-PRF**	7.679 (0.679) 14	9.748 (0.774) 14	9.230 (1.042) 14	5.370 (0.939) 14 14	7.878 (0.622) 14
**Peroxidase + L-PRF**	7.317 (0.566) 14	9.711 (0.733) 14	9.292 (0.440) 14	5.371 (0.896) 14	7.455 (0.744) 14
**Pepsin + L-PRF**	7.618 (0.396) 13	7.251 (1.180) 13	8.145 (0.864) 13	5.047 (1.271) 13	6.912 (0.693) 13
**CHX 0.12%**	6.280 (0.305) 14	7.895 (0.438) 14	6.916 (0.264) 14	4.621 (0.586) 14	8.097 (0.611) 14
**CHX 0.2%**	6.040 (0.330) 14	6.671 (0.336) 14	5.841 (0.367) 14	4.659 (0.373) 14	7.002 (0.437) 14
**PBS**	9.022 (0.519) 14	8.686 (0.519) 14	6.716 (0.913) 14	5.709 (0.994) 14	7.669 (0.433) 14

CHX: Chlorhexidine; L-PRF: Leucocyte- and platelet rich fibrin exudate; PBS: phosphate-buffered saline; HRP: horseradish peroxidase; SD: standard deviation; n: number of data.

Appendix 10. Effect of the tested solutions on commensal species in developing multi-species biofilm experiments (log_{10}(Geq/mL)).

Mean (SD) n	***S. gordonii***	***S. mitis***	***A. naeslundii***	***A. viscosus***	***S. oralis***	***S. salivarius***	***S. sanguinis***	***V. parvula***
**L-PRF**	9.251 (1.010) 14	6.011 (0.566) 14	6.951 (0.665) 14	6.952 (0.896) 14	6.284 (1.276) 14	5.599 (1.043) 10	6.111 (0.615) 14	10.013 (0.573) 14
**Inactive L-PRF**	9.173 (1.040) 14	5.964 (0.514) 14	7.046 (0.557) 14	7.235 (0.349) 14	6.070 (1.286) 13	5.136 (1.216) 13	6.004 (0.549) 14	9.989 (0.201) 14
**Peroxidase + L-PRF**	9.121 (1.0) 14	6.443 (0.611) 13	7.000 (0.546) 14	6.897 (0.460) 14	5.814 (1.182) 14	5.069 (1.130) 13	5.829 (0.472) 14	9.810 (0.215) 14
**Pepsin + L-PRF**	8.463 (1.228) 13	5.649 (0.874) 13	6.772 (0.826) 13	6.413 (0.236) 13	5.030 (0.842) 9	5.367 (1.379) 4	5.700 (0.566) 13	9.003 (0.296) 13
**CHX 0.12%**	6.362 (1.643) 14	5.901 (0.238) 14	5.557 (0.357) 14	4.737 (0.459) 14	4.867 (0.453) 10	4.734 (1.244) 0.3	4.818 (0.277) 14	8.104 (0.274) 14 14
**CHX 0.2%**	5.952 (1.545) 14	5.614 (0.220) 14	5.294 (0.332) 14	4.420 (0.292) 14	4.135 (0.592) 11	5.781 (-) 1	5.058 (0.401) 14 14	7.140 (0.201) 14
**PBS**	8.844 (0.871) 14	6.103 (0.446) 14	7.391 (0.523) 14	7.153 (0.329) 14	5.117 (0.812) 3	5.003 (1.094) 13	5.998 (0.477) 14	9.947 (0.182) 14

CHX: Chlorhexidine; L-PRF: Leucocyte- and platelet rich fibrin exudate; PBS: phosphate-buffered saline; HRP: horseradish peroxidase; SD: standard deviation; n: number of data.
